# Dietary choline intake in European and non-european populations: current status and future trends—a narrative review

**DOI:** 10.1186/s12937-024-00970-0

**Published:** 2024-06-28

**Authors:** Ewelina Zuk, Grzegorz Nikrandt, Agata Chmurzynska

**Affiliations:** Department of Human Nutrition and Dietetics, Poznań University of Life Sciences, Wojska Polskiego 31, Poznań, 60-624 Poland

**Keywords:** Choline intake, Egg and meat intake, Plant-based products, Vegetarian diet

## Abstract

**Background:**

Choline is a nutrient necessary for the proper functioning of the body with a multidimensional impact on human health. However, comprehensive studies evaluating the dietary intake of choline are limited. The aim of this narrative review is to analyze current trends in choline intake in European and non-European populations. The secondary aim was to discuss possible future choline trends.

**Methods:**

The search strategy involved a systematic approach to identifying relevant literature that met specific inclusion criteria. Observational studies and randomized clinical trials were searched for in PubMed and Scopus databases from January 2016 to April 2024. This review includes the characteristics of study groups, sample sizes, methods used to assess choline intake and time period, databases used to determine intake, choline intakes, and the main sources of choline in the diet. The review considered all population groups for which information on choline intake was collected.

**Results:**

In most studies performed in Europe after 2015 choline intake did not exceed 80% of the AI standard value. The mean choline intake for adults in different European countries were 310 mg/day, while the highest value was reported for Polish men at 519 mg/day. In non-European countries, mean choline intakes were 293 mg/day and above. The main reported sources of choline in the diet are products of animal origin, mainly eggs and meat. The available data describing the potential intake of these products in the EU in the future predict an increase in egg intake by another 8% compared to 2008–2019 and a decrease in meat intake by about 2 kg per capita from 2018 to 2030.

**Conclusions:**

In the last decade, choline intake among adults has been insufficient, both in Europe and outside it. In each population group, including pregnant women, choline intake has been lower than recommended. Future choline intake may depend on trends in meat and egg consumption, but also on the rapidly growing market of plant-based products. However, the possible changes in the intake of the main sources of choline may lead to either no change or a slight increase in overall choline intake.

## Introduction

Choline is an organic chemical compound that contains a quaternary amino group; it is also an important nutrient involved in human metabolism. The role of choline in the body has been well documented. It has different functions that depend on its chemical form [1]. First, choline is a precursor of acetylcholine, which is involved in the transmission of nerve impulses. After oxidation to betaine, which occurs mainly in the liver and kidneys, it also becomes a donor of methyl groups used in the methylation of many chemical compounds, with DNA and proteins among them [2]. Importantly, choline metabolism is closely related to the methionine cycle, which also involves several B vitamins, including folate. These pathways cross at homocysteine methylation and methionine synthesis. For this reason, insufficient supply of folate or methionine affects choline metabolism and risks choline deficiency [3, 4]. Another form of choline is phosphatidylcholine, which is a part of cell membranes responsible for their fluidity and permeability, and which also ensures the proper conduction of signals between cells. Moreover, phosphatidylcholine is essential in the synthesis of very low density lipoproteins (VLDL) and in the export of triacylglycerols from the liver [5].

Choline can be synthesized endogenously or ingested with food. De novo synthesis occurs via the hepatic phosphatidylethanolamine N-transferase (PEMT) pathway which, however, is insufficient to meet biological requirements for choline [6]. In foods, choline is found in both lipid-soluble forms (phosphatidylcholine and sphingomyelin) and water-soluble forms (free choline, phosphocholine, and glycerophosphocholine). The most important sources of choline in food are eggs, meat, fish, and whole grains. Based on the data contained in the United States Department of Agriculture (USDA) database, which includes information on the amount of choline in various products, it can be seen that total choline content is greater per unit of weight in food of animal origin than in plant-derived products [7]. The following products have the highest total choline levels per 100 g: beef liver (418 mg/100 g), chicken liver (290 mg/100 g), eggs (251 mg/100 g), wheat germ (152 mg/100 g), bacon (125 mg/100 g), raw soybean (116 mg/100 g), and pork (103 mg/100 g), while lower amounts are found in various fruits and vegetables, such as apples (3.44 mg/100 g), avocados (14.18 mg/100 g), tomatoes (6.74 mg/100 g), carrots (8.79 mg/100 g), and onions (6.10 mg/100 g) [37]. The range of food products available in Europe has changed recently, with the popularity of plant-based diets and plant-origin products growing enormously [15]. Products of animal origin nonetheless continue to represent important dietary sources of choline [9, 14].

The USDA database mentioned above was published in 2008. Since its first edition, this database has been continuously updated [7]. Most studies estimated choline intake using this database. There are at present insufficient data to assess the consequences of improper choline consumption in the population, so norms of choline intake with the diet were set at the level of adequate intake (AI). The National Academy of Medicine in the United States set a level of 550 mg/d for men and 425 mg/d for women, while the European Food Safety Authority (EFSA) in 2016 set a level of 400 mg/d for adults. The EFSA Panel on Dietetic Products, Nutrition and Allergies also noted that there is a need for more widespread measurement of choline content in food on a European level [8, 9]. Endogenous synthesis of phosphatidylcholine is not always able to provide adequate amounts of choline, and for this reason choline deficiency in the diet leads to the accumulation of lipids in the liver and non-alcoholic fatty liver disease [10, 11].

Choline is also a key nutrient for pregnant women, contributing to the normal development of the fetus. The demand for choline during pregnancy increases, and its adequate supply with the diet enables proper cell proliferation, neurogenesis, and formation of the brain and neural tube. Increased choline intake during pregnancy may thus improve neurocognitive outcomes in the offspring. However, further randomized controlled trials are needed to confirm the effects of choline in the prenatal period. The results of studies on the mutual influence of choline and folic acid/folate intake during pregnancy are particularly interesting [12, 13].

Considering the important roles played by choline in the human body, it is reasonable to monitor the dietary intake of this nutrient in populations. The aim of our review was thus to examine choline intake over the last decade, intake in European and non-European populations, in the general population as well as in specific subgroups, such as obese people and pregnant women. The secondary aim was to discuss potential future trends in choline intake.

## Methods

### Search strategy

The search strategy involved a systematic approach to identify relevant literature that met specific inclusion criteria. Observational studies and randomized clinical trials were searched for in the PubMed and Scopus databases from January 2016 to April 2024 using the following keywords: “choline” AND “intake” OR “consumption”. This resulted in 1605 citations. Only observational studies and secondary analyses of randomized controlled trials that provided information about the time period of the data collection and the method by which dietary choline intake was estimated, and which measured mean or median dietary choline intake in specific populations or study groups, were included in the final analysis. After applying these inclusion criteria, a total of 34 studies were identified, including 13 and 21 from European and non-European countries, respectively (Table 1).

**Table 1:** Studies of choline intake in European and non-European countries released after 2015

First author, country,	Study design, cohort name	Age, years, sex	Sample size	Method / Time period	Database used to determine intake	Choline intake (mg/day)	Main sources of choline	
Adults								
Redruello-Requejo et al., (2021), Spain [27]	ANIBES, cross-sectional	Female, 18–30 years Female, 31–45 years	251 390	DR x3, September to November 2013	USDA choline database	292 (236–363), median (interquartile range) 312 (256–378.), median (interquartile range)	Meat and meat products, eggs, milk and dairy products, cereals and derivatives,	
Rossi et al., (2023), Italy [66]	Case-control	Females and males	4154	FFQ, 1994–1996	USDA choline database and other available databases	401 (135), mean (SD)	Meat, cereals, poultry, eggs, vegetables, fruits and fish	
Karlsson et al. (2024), Sweden [54]	Västerbotten Intervention Programme, cohort	Females Males	52 246 50 485	semi-FFQ, 1996	USDA choline database	255 (48.6), mean (SD) 278 (55.4), mean (SD)	Not identified	
Van Parys et al., (2022), Norway [28]	Hordaland Health Study (HUSK), cross-sectional	Female, elderly Male, elderly Female, middle-aged Male, middle-aged	1539 1247 1700 1260	FFQ, 1997–1999	USDA choline database	266 (189, 382) geometric mean (95% PI) 258 (167, 390) geometric mean (95% PI) 258 (176, 380) geometric mean (95% PI) 256 (154, 402) geometric mean (95% PI)	eggs, low-fat milk, potatoes, leafy vegetables	
Van Parys et al. (2021), Norway [17]	Western Norway B Vitamin Intervention Trial (WENBIT), patients with stable angina pectoris	Female, adult Male, adult	390 1539	FFQ, 1999–2004	USDA choline database	294 (216, 435), geometric mean (95% PI) 285 (178, 439), geometric mean (95% PI)	Fish, dairy, vegetables, eggs, meat	
Muzsik-Kazimierska et al. (2022), Poland [26]	Case-control study	Postmenopausal female with HSI < 36 Postmenopausal female with HSI > 36	54 63	DR x3, 2016–2018	USDA choline database	239 (75), mean (SD) 284 (205), mean (SD)	Not identified	
Mlodzik-Czyzewska et al. (2022), Poland [18]	Case-control study	Female, 20–40 years Male, 20–40 years	207 214	DR x3, 2016–2018	USDA choline database	376 (148), mean (SD) 519 (280), mean (SD)	Eggs, poultry, pork	
Malinowska et al. (2017), Poland [24]	Secondary analysis of clinical trial outcomes (short-term folic acid supplementation)	Female, elderly	122	FFQ, 2013	USDA choline database	392 (26), mean (SD)	Not identified	
Qasrawi et al. (2024), Palestine [58]	Cross-sectional	Females	755	24 h DR x3, 2021	Eastern Mediterranean Region Food Information Data Bank	136 (65) mean (SE)	Not identified	
López-Carrillo et al. (2016), Mexico [23]	Cross-sectional	Female, > 20 years	1027	FFQ, 2007–2011	Data not provided	263 (105), mean (SD)	Not identified	
Probst et al. (2019), Australia [36]		National Nutrition and Physical Activity Survey (NNPAS), Australian Health Survey	Female, 19–64 years Male, 19–64 years	3640 3372	Dietary recall, 2 days, 2011–2013	Australian choline database based on USDA choline database and other studies	248 (2), mean (SE) 311 (2), mean (SE)	Eggs, dairy milk (cow, sheep and coat) unprocessed and processed meat
Gao et al. (2016), Canada [21]	Complex Disease in Newfoundland population: Environment and Genetics (CODING) study, cross-sectional	Female, normal BMI, adult Male, normal BMI, adult	1021 234	Willet FFQ, 2003–2015	NutriBase Clinical Nutrition Manager (v. 8.2.0; Cybersoft, Phoenix, AZ)	299 (221), mean (SD) 423 (327) mean (SD)	Not identified	
Cheng et al. (2017), Taiwan [16]	Cross-sectional	Male > 20 years Female > 20 years	321 227	qFFQ, 2002–2009	USDA choline database and other analytical reports	284 (145), mean (SD) 230 (120), mean (SD)	Not identified	
Golzarand et al. (2022), Iran [22]	Tehran Lipid and Glucose Study, cohort	Female, male, adults	2606	FFQ, 2006–2008	USDA choline database	226 (223–228), geometric mean (95% PI)	Meat, grains and bakery products, dairy products	
Wallace (2016), USA [19]	National Health and Nutrition Examination Survey (NHANES), cross-sectional	Female, 19–30 years Male, 19–30 years	1170 1096	Dietary recall, 2 days, 2008–2012	USDA National Nutrient Database for Standard Reference	250 (5), mean (SE) 387 (7), mean (SE)	Not identified	
Wallace (2017), USA [20]	National Health and Nutrition Examination Survey (NHANES), cross-sectional	Female, 19–30 years Male, 19–30 years	1586 1702	Dietary recall, 2 days, 2005–2014	USDA National Nutrient Database for Standard Reference	257 (4), mean (SE) 392 (5), mean (SE)	Eggs, meat, poultry	
Millard et al. (2018), USA [25]	Jackson Hearth Study (JHS)	African-American females, 21–94 years African-American males, 21–94 years	2613 1311	FFQ, 2000–2004	USDA food content databases	278 (126), mean (SD) 357 (147), mean (SD	Non-fried eggs, fried fish, corn bread or muffins, fried beef, whole milk	
Freedman et al. (2024), USA [56]	National Health and Nutrition Examination Survey (NHANES), cross-sectional	Males Females	2365 2498	24 h DR, 2017–2018	USDA Food and Nutrient Database for Dietary Studies	385 (4), mean (SE) 280 (7), mean (SE)	Not identified	
Zhou et al. (2023), USA [63]	National Health and Nutrition Examination Survey (NHANES), cross-sectional	Females and males aged > 20 years old	14 323	24 h DR x2, 2011–2016	USDA Food and Nutrient Database for Dietary Studies	317 (164), mean (SE)	Not identified	
Sheyn et al. (2024) [64], USA	Nurses Health Study I Nurses Health Study II	Females	8318 9 684	FFQ, 1980 FFQ, 1991	USDA choline database USDA choline database	304 (28) mean (SD) 312 (32) mean (SD)	Not identified	
Zhang et al. (2024) [65], USA	NHANES	Females and males aged ≥40 years	660	Dietary recall, 2 days, 2013–2014	Food and Nutrient Database for Dietary Studies	218, mean	Not identified	
Pregnant women								
Pauwels et al. (2017), Belgium [33]	The Maternal Nutrition and Offspring’s Epigenome (MANOE) study, observational cohort study	Pregnancy, first trimester	94	FFQ, April 2012 - March 2016	USDA choline database	274 (7), mean (SE)	Not identified	
Roeren et al. (2022), Germany [13]	Cross-sectional	Pregnancy	273	FFQ, November and December 2021	USDA choline database	260 ± 141, median	eggs, red meat, white meat for omnivores eggs, green kale and fruit juices for vegetarians/vegans	
Moore et al. (2020), United Kingdom [35]	The Be Healthy in Pregnancy (BHIP) study, baseline data of RCT	Pregnancy	232	DR x3, 2012–2018	Canada choline database	338 (120, 1016), median (min, max)	Meat and fish, eggs, milk and alternatives, vegetables and fruits	
Chmurzynska et al. (2018), Poland [34]	Cross-sectional	Pregnancy, third trimester	74	FFQ, 2016	USDA choline database	365 (14), mean (SD)	Eggs, poultry	
Staskova et al. (2023), Australia [60]	Barwon Infant Study, cohort	Pregnancy	236	Dietary Questionnaire for Epidemiological Studies Version 2, 2010–2013	USDA choline database	372 (104), mean (SD)	Dairy, eggs, bakery, vegetables, beef, chicken and turkey	
Robb et al. (2023), South Africa [59]	The Nuemi Study, cross-sectional	Pregnancy	682	qFFQ, 2018–2019	USDA choline database	275 (85–387), mean (interquartile range)	Not identified	
Wiedeman et al. (2024), Canada [61]	CHILD, cohort	Pregnancy	2996	Semi-FFQ, 2009–2012	USDA choline database	375 (151), mean (SD)	Not identified	
Probst et al. (2019), Australia [36]	National Nutrition and Physical Activity Survey (NNPAS), Australian Health Survey	Pregnancy	116	Dietary recall, 2 days, 2011–2013	Australian choline database based on USDA choline database and other studies	253 (10), mean (SE)	Eggs, dairy milk (cow, sheep and coat) unprocessed and processed meat	
Christifano et al. (2022), USA [32]	Cross-sectional	Pregnancy, 32 week	202	DHQ-II Diet History Questionnaire II	Diet*Calc analysis software	275 (138), mean (SD)	Not identified	
Agarwal (2024), USA [55]	National Health and Nutrition Examination Survey (NHANES), cross-sectional	Pregnancy and lactation	319	24 h DR, 2011–2018	USDA Food and Nutrient Database for Dietary Studies	314 (9), mean (SE)	Not identified	
Olendzki et al. (2023) [57], USA	Case-control	Pregnant females with IBD Pregnant females without IBD	88 82	24 h dietary recall, January 2019- December 2022	USDA choline database	360 (146) mean (SD) 354 (129) mean (SD)	Not identified	
Children and adolescents								
Prelicz (2017), Romania [31]	Cross-sectional	Boys, 4–6 years Girls, 4–6 years	34 37	DR x3, September–October 2011	USDA choline database	214 ± 26, mean ± SD 215 ± 37, mean ± SD	Meat (mainly poultry), eggs, grains, cereals, baked products, dairy products	
Probst et al. (2019), Australia [36]	National Nutrition and Physical Activity Survey (NNPAS), Australian Health Survey	Children, 2–3 years	464	Dietary recall, 2 days, 2011–2013	Australian choline database based on USDA choline database and other studies	185 (3), mean (SE)	Eggs, dairy milk (cow, sheep and coat) unprocessed and processed meat	
Jafari et al. (2021), Iran [29]	Cross-sectional	Girls, 6 years	788	FFQ, 2017–2018	USDA food content databases	263 (137), mean (SE)	Not identified	
Wallace (2016), USA [19]	National Health and Nutrition Examination Survey (NHANES), cross-sectional	Girls, 2–3 years Boys, 2–3 years	485 471	Dietary recall, 2 days, 2008–2012	USDA National Nutrient Database for Standard Reference	216 (6), mean (SE) 241 (7), mean (SE)	Not identified	
Wallace (2017), USA [20]	National Health and Nutrition Examination Survey (NHANES), cross-sectional	Girls, 2–3 years Boys, 2–3 years	658 658	Dietary recall, 2 days, 2005–2014	USDA National Nutrient Database for Standard Reference	201 (4), mean (SE) 246 (5), mean (SE)	Eggs, meat, poultry	
Zhang et al. (2024), USA [62]	National Health and Nutrition Examination Survey (NHANES), cross-sectional	Children and adolescents aged 2–17 years old	4715	24 h DR, 2015–2018	USDA Food and Nutrient Database for Dietary Studies	248 (3), mean (SE)	Not identified	

Abbreviations: DR: dietary record; FFQ: Food Frequency Questionnaire; SE: standard error; SD: standard deviation; USA: United States of America; USDA: United States Department of Agriculture; qFFQ : quantitative Food Frequency Questionnaire

## Results

### Current trends in choline intake

As a growing number of studies confirms the important role of choline in human nutrition, it is crucial to estimate the intake of this nutrient in various populations. It should be noticed that the great majority of the identified studies released after 2015 are based on data obtained in previous decades (1992–2015), and cannot be relied on in discussing current trends in choline intake. The studies were conducted in nine European countries only: Poland [18, 24, 26, 34], Norway [17, 28], Germany [13], Spain [27], Sweden [54] Romania [31], Belgium [33], Italy [66] and the United Kingdom [35]. Eight studies have reported choline intake in adults [17, 18, 24, 27, 28, 54, 66], one study measured choline intake in children [31], and four studies have recorded choline intake in pregnant women [13, 33–35]. Among the twenty-one studies measuring choline intake in non-European countries, the majority (52%) were conducted in the USA [19, 20, 25, 32, 55–57, 62–65], while the others were carried out in Palestine [58], South Africa [59], Australia [36, 60], Mexico [23], Taiwan [16], Iran [22, 29], Canada [21, 61].

Thirteen studies identified the main sources of choline as being mainly products of animal origin, such as eggs, meat, dairy, dairy derivatives, and fish, as well as grain and bakery products [12, 13, 17, 18, 20, 23, 25, 27, 28, 31, 34, 35, 60, 66], although not every study provided detailed data (Table 1).

### Choline intake in adults

We identified twenty one studies that measured choline intake in adults in European and non-European countries. Four out of these were based on data obtained after 2015, and reported the mean intake at 317 mg/day. The mean intake of choline in the European countries was 310 mg/day. Only two of the eight studies of European adults showed adequate choline intake, as measured by EFSA recommendations [18, 66]. Five studies showed choline intake in adult females and males to be less than 80% of the AI established by the EFSA [17, 26–28, 54]. Choline intake was relatively high in healthy Polish men aged 20–40 (519 ± 280 mg/day). This may have resulted from different dietary patterns. Interestingly, the contribution of eggs in total choline intake was 33% in Polish subjects, while in most studies this did not exceed 20% [18]. Another Polish study conducted after 2015 showed inadequate choline intake in postmenopausal women [26].

The mean choline intake among adults in non-European countries was 293 mg/day. A study conducted in Canada showed choline intake above the EFSA AI in normal-weight men (422.85 ± 326.87 mg/day), but not in overweight or obese men (358.08 ± 267.35 mg/day and 341.49 ± 268.27 mg/day, respectively). The difference between normal-weight and overweight/obese men and women was statistically significant when choline intake was considered as mg/kg of body mass/day. The decline in choline intake was 36% in obese women and 44% in obese men comparing to normal weight [21]. Contrary to those results, a study conducted in Poland did not show differences in choline intake between normal weight and overweight/obese people. The study conducted by Wallace et al. [20] in the United States showed that eggs and protein food (meat, seafood) are the major sources of choline in the diet. Interestingly, adults who consume eggs were more likely to meet the AI requirements than nonconsumers of eggs (57.3% ± 1.45% vs. 2.43% ± 0.28% respectively). Additionally, the intake of choline in the egg consumers was almost twice that of nonconsumers (525 ± 5.17 mg/d vs. 294 ± 1.98; *p* ≤ 0.0001).

### Choline intake in pregnant women, children, and adolescents

The studies of pregnant women showed low levels of choline intake ranging from 260.4 mg/day to 372 mg/day [13, 32–36, 57, 59–61]. In a study conducted by Chmurzynska et al. in a group of 74 pregnant women in Poland, only 27% of participants achieved an adequate intake of choline (AI = 450 mg/d) [34]. Interestingly, the study of Roeren et al. [13] in Germany showed a statistically significant difference in the choline intake of pregnant women who were vegetarians or vegans and those who were omnivores (205.2 mg/day vs. 269.5 mg/day respectively). The vegetarian/vegan group had a 30% lower odds ratio for meeting the choline AI than the omnivorous group. Moreover, only 5% of participants took dietary choline supplements, which provided 19% of choline intake [13]. However, 95% of the vegetarians/vegans and 93% of the omnivores had inadequate choline intake. To the best of our knowledge, thus was the first study to assess choline intake in vegans and vegetarians. Another study conducted in Canada showed that 11% of pregnant woman took supplements that contributed to 34% of total choline intake. Only 18% of women met the AI for choline [35]. Similarly, Staskova et al. [60] showed that 23% of pregnant Australian females had adequate choline intake and that 2.8% of participants took choline supplements. In another Australian study, 75% of woman in early pregnancy and 67% of woman in late pregnancy did not receive adequate choline intake by the EFSA recommendations. It should be noted, however, that these data were obtained in a randomized-controlled trial focused on folic acid supplementation [17]. That study identified eggs, red meat, and legumes as the three most important sources of choline, while the German study did not identify legumes as a source of choline in the diet of pregnant women [13].

Choline intake was found to be above AI values only in infants and children aged 2–6 years [18, 19, 29–31, 36].

In summary, most of the studies published in recent years have been based on out-of-date data. Choline is essential nutrient, especially in fetal neurodevelopment, so it is crucial to re-evaluate our knowledge of its current intake in European countries. Moreover, the development of a European choline database should be a future research direction.

## Discussion

### Current choline intake in Europe and outside Europe

Choline intake appears to be insufficient for various population groups across the world. Moreover, there has been very few studies to analyze the consumption of individual forms of choline [2]. The available data show that the lipid-soluble forms of choline (such as phosphatidylcholine) account for about half of the total amount of choline consumed by the European population [17]. In the vast majority of studies describing European and non-European countries, the main sources of choline were products of animal origin, including eggs, meat, milk; these contain choline primarily in the form of phosphatidylcholine [37].

The findings from our review align with previous research conducted before 2016 and spanning the last 20 years, which shows ongoing trends in the consumption of this nutrient. The first European study focusing on estimating choline intake across different sex and age groups on the European level was conducted in 2015 by Vennemann et al. This study analyzed twelve dietary surveys representative of general populations or specific demographic groups. Overall, the study revealed inadequate choline intake across most population groups relative to the Adequate Intake (AI) values established by the National Academy of Medicine in the USA. The study collected data on choline intake across various age groups: toddlers (1–3 years) had average choline intakes ranging from 151 to 210 mg/d, which generally fell below the AI. In the six studies of choline intake in children that we examined, this was the age group that came closest to meeting the AI level. A 2015 study reported that, among males, average choline intake estimates ranged from 309 to 444 mg/d across different age groups, while among females, estimates ranged from 244 to 404 mg/d. In our review, the average choline intake among adults in EU countries was 310 mg/day, while in non-European countries it was 293 mg/day. Additionally, the 2015 review included one analysis of choline intake among pregnant women, finding that the average intake among adult pregnant women was approximately 356 mg/d. In our study, the average intake for pregnant women in both European and non-European countries ranged from 260 to 372 mg/day. The review of Vennemann et al., indicated that men generally exhibited higher choline intake than women, largely due to their higher daily food intake [14]. This trend corroborates with the findings observed in our review. The 2015 review also demonstrated results similar to ours, highlighting meat, milk, and dairy products as the most significant dietary sources of choline.

Attention should be paid to the methodological differences between the various studies, which makes it impossible to directly compare choline intake between countries. For this reason, it seems that it will be necessary to harmonize the data collection methodology on both national and global levels. This would make it easier to apply and create nutritional recommendations for particular groups of the overall population.

### Prognosis of future choline intake trends

When forecasting the level of choline intake in European populations in coming years, trends in the consumption of eggs and meat, and the popularity of vegetarian and other diets that limit the consumption of animal products should be considered.

The levels of consumption of products of animal and plant origin result from global trends that are affect both by nutritional issues (such as the need to ensure adequate food quality for increasing populations) and by aspects related to environment and climate change. An analysis of the years 2000 to 2017 confirmed that Europe has a higher rate of consumption of food of animal origin than other world regions. Taking into account two of the three scenarios described by the Food and Agriculture Organization (FAO), worldwide demand for protein of animal origin will increase in the coming years. Interestingly, the third scenario, based on the sustainable development strategy, assumes a decline in the consumption of animal products in Europe. This approach involves the promotion of diets based primarily on products of plant origin in European countries [38]. This has been described in the EAT–Lancet report from 2019 and in the European Union’s 2020 Farm to Fork strategy [38, 39]. Our analysis will consider the consumption of eggs and meat separately, as studies show differences in the impact of these products on human health.

A closer look at the data on egg consumption in European Union countries shows that it increased by 4% in 2008–2019 and that it is expected to increase by another 8% by 2030. Data prepared by the FAO in 2020 also show that the annual consumption of eggs in most EU member states equals or exceeds the global average. Importantly, there are differences in egg consumption between EU Member States. It can be assumed that this is related to different nutritional recommendations for egg consumption depending on the country. For example, there are no limits on the consumption of eggs in the United Kingdom, where they are considered a “healthy product”. As mentioned earlier, eggs among the main sources of choline. If the above predictions come true, eggs will remain an important food product that provide significant amounts of dietary choline. The trend towards not limiting egg consumption may also be due to the fact that several studies have positively associated egg consumption with diet quality and a greater likelihood of meeting the recommended intake of vitamins and minerals [40–42]. The EAT–Lancet Committee on Healthy Diets recommended eating 1.5 eggs per week, but the report concluded that higher egg consumption may benefit those on low-quality diets, especially among low-income populations [39]. It is also worth mentioning that the American Heart Association and the 2015–2020 Dietary Guidelines for Americans removed the 300 mg/d limit for dietary cholesterol, without specifying a new recommended level of dietary cholesterol intake. However, it has been established that healthy people are advised to consume a maximum of one whole egg per day as a part of a healthy diet. These are general recommendations that are intended to form part of the promotion of healthy dietary patterns (e.g., the Mediterranean diet) [43].

A European Commission report suggests that the overall per capita consumption of meat in EU countries will fall to 67 kg by 2031, from a level of 69.8 kg in 2018. The decrease will mainly be in red meat. Consumption of beef is forecasted to decrease from 10.6 kg to 9.7 kg per capita in 2021–2023. The consumption of pork is also expected to fall to 31 kg per capita in 2031, from a level of 32.5 kg in 2021. The reasons for this can be found in changes in the food preferences of consumers in the EU, which are associated to increasing awareness of the impact of diet on health and the environment. A different situation is forecasted for poultry, which is currently considered healthier than other types of meat, with the consumption of poultry expected to increase from 23.5 kg per capita in 2021 to 24.8 kg in 2031. Interestingly, a slight increase in the consumption of sheep meat is also expected, which is expected to reach 1.4 kg per capita by 2031 [44]. Summarizing this part, it is likely that the overall consumption of meat in the European Union countries will decrease in the coming years, but that this decrease will not be significant. It can therefore be assumed that, as in the case of eggs, meat will remain one of the main sources of choline in the diet.

The increasing popularity of vegetarian diets may significantly affect choline intake. However, there has been only one study to date that has compared choline intake in vegetarians and nonvegetarians. It has been shown that pregnant women on a vegetarian diet consume statistically significantly less choline than omnivorous women [13]. This small amount of data leaves open the question of whether people on vegetarian diets can meet their choline needs.

Alcorta et al. collected data on the growing number of people choosing a vegetarian diet (in its various variants). The tendency to increase the consumption of plant-derived products at the expense of animal products is noticeable in both the United States and in European countries. What is more, a 2019 global study found that 40% of consumers try to limit their animal protein intake, while 10% avoid red meat altogether. The practice of occasionally eating food from animals while otherwise maintaining a vegetarian diet, referred to as flexitarianism, has also became popular [15, 45]. As mentioned, the popularity of vegetarian diets is due to increasing evidence of their positive effects on health. However, it should not be forgotten that an unbalanced plant diet based on highly processed products can be deficient in many nutrients, including choline [46, 47]. Recent reports have emphasized that recommending plant-based diets does not have to be associated with complete avoidance of animal products, but only limiting their quantity in favor of plant-based products [38]. The trend of reducing the consumption of animal products is also due to environmental concerns, as vegetarian diets use less natural resources and are associated with significantly less environmental damage [48].

Meat and egg substitutes should be considered in the context of vegetarian diets. Meat substitutes are products that should be similar in terms of taste, nutritional, and aesthetic values to various types of meat. They are generally prepared from soybeans, but sometimes also from other legumes, nuts, cereal proteins, and vegetables. There is also great potential for new meat substitute ingredients in algae, yeast, and fungi. The overriding goal in the production of substitutes is to achieve the highest possible biological value of the protein and to enrich monotonous diets based on vegetable protein [49]. Soy is the main material of interest for improving choline content in vegetarian diets employing meat substitutes, as it is a good source of choline with 116 mg of total choline in 100 g. Soy is considered to be one of the best plant sources of choline in the diet [37, 50]. However, one major limitation affecting the analysis of choline in meat and egg substitutes is the very limited data available on the subject. The USDA database contains 634 food products for which the content of betaine, various forms of choline, and total choline have been described. This database is a source for the content of choline in various products, but unfortunately is not updated on an ongoing basis and does not include new products that are plant-based substitutes for animal products. The database indicates that legumes contain an average of 49 mg of total choline per 100 g [7, 51]. According to the Canadian Food Guide, consuming legumes alone would cover 20% of choline requirements [51]. However, it should also be mentioned that not all meat substitutes will provide choline, as it depends on the raw materials they are made of [49].

The most commonly used ingredients in egg substitutes are vegetable proteins, fiber, polysaccharides, lecithin, flavoring and coloring additives, and multi-ingredient mixtures containing the above ingredients in given proportions [52]. The literature discussion of egg substitutes mainly concerns technical aspects and their use to affect the structure of a product. For example, Wang et al. examined the use of soybean oil as an egg substitute in the preparation of a vegetarian version of mayonnaise [53]. Unfortunately there is no data on the content of choline in ready-made substitutes, and instead their ingredients must be analyzed - however, this ignores the effects of the production process on choline content [51].

Plant-based meat and egg substitutes are seen as alternatives capable of reducing the consumption of animal products with all the effects of such reduction, including their positive impact on health. However, we cannot ignore the fact that some of these products are highly processed, with high sodium contents and unbalanced fatty acid profiles. Consumers should thus be advised to carefully consider the composition of such products before using them. More research is also needed on their nutritional value and on the content of individual nutrients, such as choline.

## Conclusions

In summary, data from the past decade reveal a dearth of research delineating choline intake within diverse populations and dietary contexts. There is a noticeable absence of comprehensive summaries akin to our review, underscoring the need for more research in this area. Since this is an important nutrient for human health, it becomes necessary to create a database of products containing choline, taking into account locally occurring products, as well as conducting extensive research to determine the current choline dietary standards in individual age groups. It would also be justified to conduct studies on choline intake with dietary supplements, as well as analyze what supplements with this ingredient are available on the European market. However, it is at first necessary to determine the EAR and RDA levels for choline because this would enable a realistic assessment of the coverage of the demand for choline. Analyzing potential trends in consumption, we can predict that the next 5–10 years will probably increase egg consumption, which can contribute to higher choline consumption. On the other hand, trends and nutritional recommendations are currently focused on limiting meat consumption and promoting vegetarian diets. Together, these could lead either to no change or to a slight increase in the consumption of choline, which already seems inadequate in many countries. Responding to this tendency will require great efforts in public health and nutritional education focusing on the meaning of choline for the general public. In addition to more familiar forms of education—such as lectures and culinary workshops for the target age groups—the creation of content on social media dealing with the importance of dietary choline intake for health may also prove helpful in conducting effective campaigns. The short format of this type of message makes it capable of reaching a larger group of recipients, especially in younger age categories, which can help build healthy eating habits at an earlier stage of life. Taking account of trends in communication when educating the public is an essential means of using the results of scientific research to produce real change.

## Data Availability

Not applicable.

## Abbreviations

EAR: Estimated average requirement
AI: Adequate intake
EU: European Union
VLDL: Very low density lipoproteins
PEMT: Phosphatidylethanolamine N-transferase
USDA: United States Department of Agriculture
EFSA: European Food Safety Authority
FAO: Food and Agriculture Organization
